# Pamphlets of the Month

**Published:** 1855-06

**Authors:** 


*Pamphlets of the Month.*—The Smithsonian Institution has favored us—duly franked—with the "Report of Hon. Wm. H. White, from the Committee of the House of Representatives," to which was referred the letter of Hon. Rufus Choate, on the Smithsonian Institution.

The Hon. Wm. H. White, we conclude, must be the gentleman who made a minority report in favor of the Institution. The arguments are, strictly speaking, arguments only; the facts and evidence being excluded from the report. We have seen nothing as yet to alter our conviction that the Smithsonian Institution, under its present management, is a scientific bagatelle, arbitrarily controlled by a single man for the benefit of himself and satellites.

*On the Clinical Analysis of the Tennessee Collection of Urinary Calculi.* By E. B. Haskins, M. D.

Dr. Haskins has here given the analysis of 180 calculi, of which all but four were from the human subject. The results obtained contradict those of other localities, free uric acid being the predominant constituent in only four out of 176. The urates of ammonia, lime, and soda, are the principal constituents of the calculi examined by Dr. Haskins. The pamphlet evidences much care and patient research on the part of its author.

*Constitution und Nebengsetze des deutschen Medizinischen Vereins der Stadt, Buffalo.*

The German physicians of this city, have organized themselves into a society of which the constitution is before us. This action is taken with a view of separating themselves from the rabble of German quacks with whom they are necessarily more or less associated in the minds of the community. To obtain membership in this society, the candidate must present full evidence that, previous to his emigration, he was a reputable and legally authorized practitioner. We are glad to notice that several German practitioners, having American educations and diplomas, are engaged in this effort.

At the last meeting of the Erie County Medical Society, a committee was appointed to confer with the German society as to the most ready means of affiliation.

